# A Jacalin-Related Lectin Regulated the Formation of Aerial Mycelium and Fruiting Body in *Flammulina velutipes*

**DOI:** 10.3390/ijms17121884

**Published:** 2016-11-28

**Authors:** Yuan-Ping Lu, Ren-Liang Chen, Ying Long, Xiao Li, Yu-Ji Jiang, Bao-Gui Xie

**Affiliations:** 1 Mycological Research Center, College of Life Sciences, Fujian Agriculture and Forestry University, Fuzhou 350002, China; yuanplu1106@163.com (Y.-P.L.); renlchen2306126618@163.com (R.-L.C.); yinglong695489624@163.com (Y.L.); 18259088174@163.com (X.L.); 2 College of Food Sciences, Fujian Agriculture and Forestry University, Fuzhou 350002, China

**Keywords:** mushroom, overexpression, RNAi, transformation

## Abstract

*Flammulina velutipes*, one of the most popular mushroom species in the world, has been recognized as a useful model system to study the biochemical and physiological aspects of the formation and elongation of fruit body. However, few reports have been published on the regulation of fruiting body formation in *F. velutipes* at the molecular level. In this study, a jacalin-related lectin gene from *F. velutipes* was characterized. The phylogenetic tree revealed that Fv-JRL1 clustered with other basidiomycete jacalin-like lectins. Moreover, the transcriptional pattern of the *Fv-JRL1* gene in different developmental stages of *F. velutipes* implied that *Fv-JRL1* could be important for formation of fruit body. Additionally, RNA interference (RNAi) and overexpression analyses provided powerful evidence that the lectin gene *Fv-JRL1* from *F. velutipes* plays important roles in fruiting body formation.

## 1. Introduction

Lectins are non-immunoglobulin proteins and that are widely distributed among organisms, including animals, plants, and microorganisms [1,2], in which they perform a variety of functions. In plants, most lectins act as a defensive system against fungi and insects [3,4,5,6], and some lectins mediate the symbiotic relationship of leguminous plants and nitrogen-fixing bacteria [7]. Animal lectins play a regulative role in organ differentiation and formation [8], and participate in metastasis of cancer cells [9,10] as well as the migration of lymphocytes from the bloodstream into the lymphoid organs [11]. In some viruses, lectins are primarily employed to attachment to host cells [2].

Mushrooms, defined as macrofungi with conspicuous fruiting structures, have been consumed by humans since earliest recorded history, due to their high nutritional properties, as well as their desirable taste and flavor [12,13]. Medicinal attributes, the second major property of mushrooms, have also been recognized and utilized by Oriental cultures, particularly in China and Japan, for a very long time [13,14,15].

Mushrooms contain large amounts of medicinal and pharmacological bioactive compounds [16,17,18,19]. Among them, mushroom lectins have captured increased attention because of their antitumor, antiproliferative, and immunomodulatory activities [20,21,22]. The edible basidiomycete *Flammulina velutipes*, one of the six most widely grown and consumed mushroom species in the world with an annual yield greater than 300,000 tons, expresses high level of lectins, approximately 77 mg of purified lectin (FVA) per 200 g of fruiting bodies [23]. However, the physiological roles of lectins from *F. velutipes* in nature are not well known. Previous studies have demonstrated that lectins from *Pleurotus cornucopia*e have been involved in the process of fruit body formation [24]. Thus, it was speculated that some lectins from *F. velutipes* may similarly be involved in formation of the fruiting body.

In this study, a jacalin-related lectin (JRL) gene from *F. velutipes*, designated as *Fv-JRL1*, was identified and characterized. The expression levels of the *Fv-JRL1* gene were also characterized in different developmental stages of *F. velutipes.* The transcriptional pattern suggested that *Fv-JRL1* could be important for formation of the fruiting body. Additionally, RNAi and overexpression were also conducted in order to study the function of this gene, and the results strongly suggest that *Fv-JRL1* from *F. velutipes* does really play an important regulatory role in formation of aerial mycelium and fruiting body.

## 2. Results

### 2.1. The Structure of the Fv-JRL1 Gene and Phylogenetic Analysis of the Fv-JRL1 Protein

The sequence of the *Fv-JRL1* gene was retrieved from the genome of *F. velutipes* strain W23. The *Fv-JRL1* gene was found to be 1126 bp, encompassing the full-length *Fv-JRL1* gene, as well as a 245 bp 5’-UTR and 106 bp 3’-UTR. Four introns of 54, 53, 66, and 59 bp, as well as highly conserved consensus sequences at the 5’ (GT) and 3’ (AG) splice junctions were identified. In addition, comparison of the sequences of *Fv-JRL1* in strains L11 and W23 using DNAMAN [25] indicated that the sequences between the two strains were identical, eliminating the interference of homologous genes at transcription level and the need for further RNA interference (RNAi) analysis.

**Figure 1:**
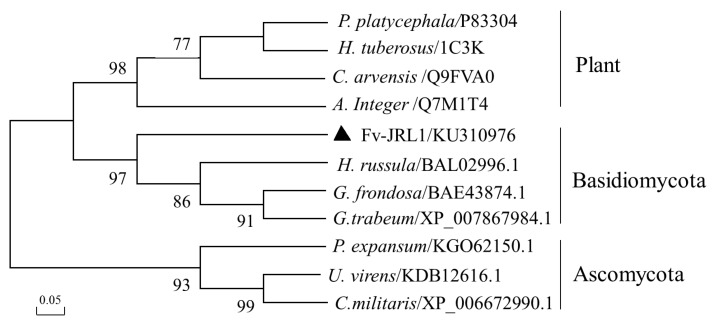
Phylogenetic analysis of Fv-JRL1 with amino acid sequences of JRL1 identified from different organisms. *P. platycephala*, *Parkia platycephala*; *H. Tuberosus*, *Helianthus Tuberosus*; *C. arvensis*, *Convolvulus arvensis*; *A. integer*, *Artocarpus integer*; *H. russula*, *Hygrophorus russula*; *G. trabeum*, *Gloeophyllum trabeum*; *G. frondosa*, *Grifola frondosa*; *P. expansum*, *Penicillium expansum*; *U. virens*, *Ustilaginoidea virens*; *C. militaris*, *Cordyceps militaris*. The triangle indicates *Flammulina velutipes* Fv-JRL1 protein.

Analysis with ExPASyProtparam (http://www.expasy.ch/tools/protparam.html) [26] indicated that the deduced Fv-JRL1 protein contained 180 amino acids and had a theoretical isoelectric point (pI) of 4.97 and a predicted molecular weight of 19.479 kDa. Four N-myristoylation sites (G^62^IQPTY, G^140^TSFGT, G^147^QVIAL, and G^154^TDENS) were identified in this protein using ExPASyProsite (http://prosite.expasy.org/). A conserved jacalin-like lectin domain (IPR001229) at amino acids 46–137 was found by InterProScan search (http://www.ebi.ac.uk/Tools/pfa/iprscan/) [27]. For phylogenetic analysis, the amino acid sequences of JRL1 proteins from different organisms were obtained from NCBI and EBI, and aligned with Clustal_X 1.83 [28]. The neighbor-joining tree (Figure 1) generated by MEGA 5.0 with 1000 replicates of bootstrap analysis [29,30] displayed that all of the jacalin-like lectins collected from plants, Basidiomycota, or Ascomycota formed independent clades, and Fv-JRL1 from *F. velutipes* and jacalin-like lectins from other Basidiomycota fungi clustered into the same clade with a well-supported bootstrap value of 97%.

### 2.2. Expression Patterns Revealed the Potential Function of Fv-JRL1 Protein in the Formation of the Fruiting Body

**Figure 2:**
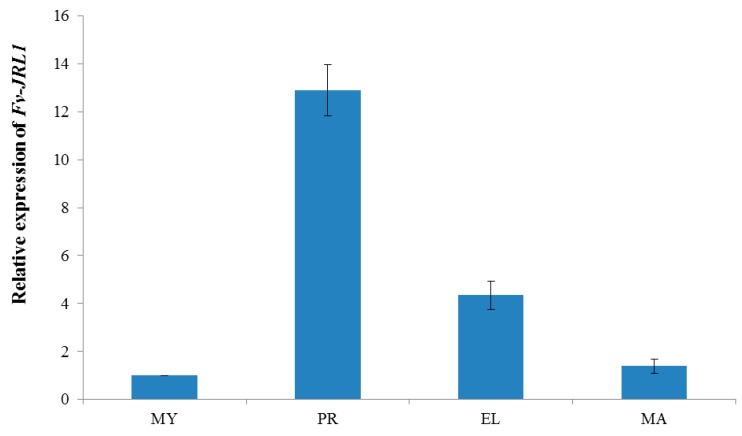
Expression patterns of *Fv-JRL1* during different development stages in *F. velutipes*. MY, mycelium; PR, primordium; EL, elongation stage; MA, mature fruit body.

To investigate the possible roles of Fv-JRL1 protein, we assessed the expression of *Fv-JRL1* gene during different development stages using qRT-PCR. The results (Figure 2) indicated that the expression of *Fv-JRL1* in the primordial stage was upregulated by approximately 13-fold relative to the mycelial stage (Figure 2). Additionally, *Fv-JRL1* transcription was decreased at the elongation and mature stages. These results suggested that Fv-JRL1 proteins are likely related to the formation of the fruiting body.

### 2.3. Confirmation of the Ratio between Plasmid DNA and Liposomes

**Figure 3:**
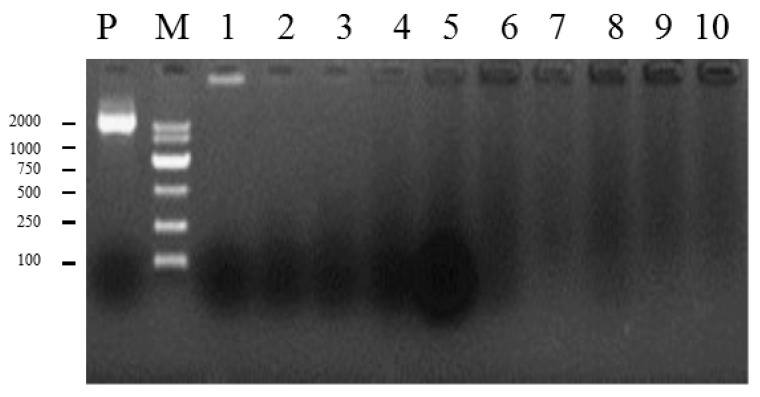
Gel electrophoresis of plasmid DNA (lane P) and plasmid DNA–liposome (lanes 1–10) composed of 1 µg plasmid DNA and 1–10 µL liposome. Lane P, naked plasmid DNA. Lane M, Marker DL2000. Lanes 1–10, 1 µg plasmid DNA mixed with 1, 2, 3, 4, 5, 6, 7, 8, 9, and 10 µL liposome, respectively.

In the present study, the efficiency of DNA encapsulation was assessed using agarose gel electrophoresis [31,32]. As shown in Figure 3, a DNA band migrated towards the cathode in lane 1, indicating plasmid DNA that was not encapsulated in liposome, as compared to lane P. Thus, the proportion (1 µg plasmid DNA: 1 µL liposomes) was chosen as the optimal ratio for transformation.

### 2.4. Generation of Mutants and Fv-JRL1 Gene Expression Analysis

After liposome-mediated transformation, colonies with hygromycin B resistance were obtained and verified by PCR. The presence of the hygromycin B phosphotransferase (*hpt*) gene (1057 bp), as well as the sequences between the glyceraldehyde-3-phosphate dehydrogenase (*gpd*) gene promotor (*Pgpd*) and the *Fv-JRL1* gene (1084 bp), was confirmed in overexpression transformants OE1 and OE14 (Figure S1). The presence of the *hpt* gene and sequences between the *Pgpd* and the first intron of *Fv-JRL1* (733 bp) were identified in RNAi transformants Ri1 and Ri2 (Figure S1).

**Figure 4:**
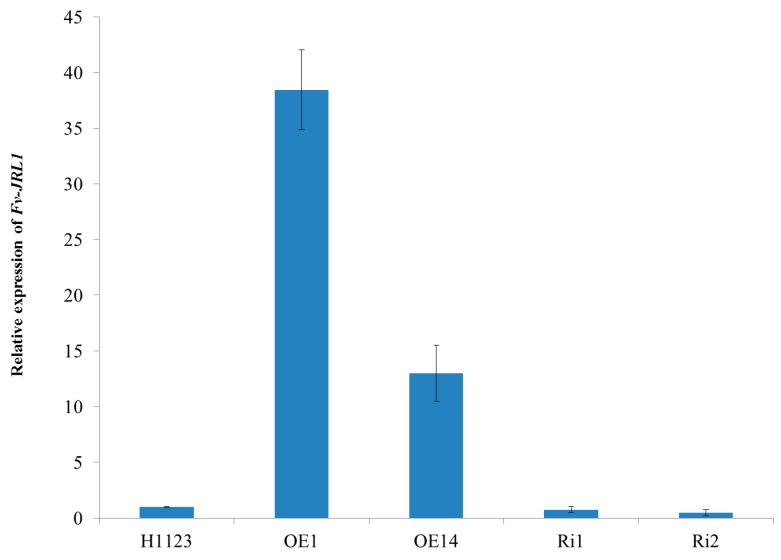
The transcription analysis of *Fv-JRL1.* The relative expression level of *Fv-JRL1* was evaluated as fold changes in comparison with the expression level in the wild-type (WT) strain H1123.

In order to verify that *Fv-JRL1* was silenced or overexpressed, the expression levels of the *Fv-JRL1* gene were measured in all transformants. Compared to the wild-type strain H1123, the expression levels of *Fv-JRL1* were upregulated approximately 35-fold and 13-fold in transformants OE1 and OE14, respectively (Figure 4). In contrast, *Fv-JRL1* gene expression was decreased by 26% and 51% in Ri1 and Ri2, respectively, compared to H1123.

### 2.5. Phenotypic Characterization of Mutants

**Figure 5:**
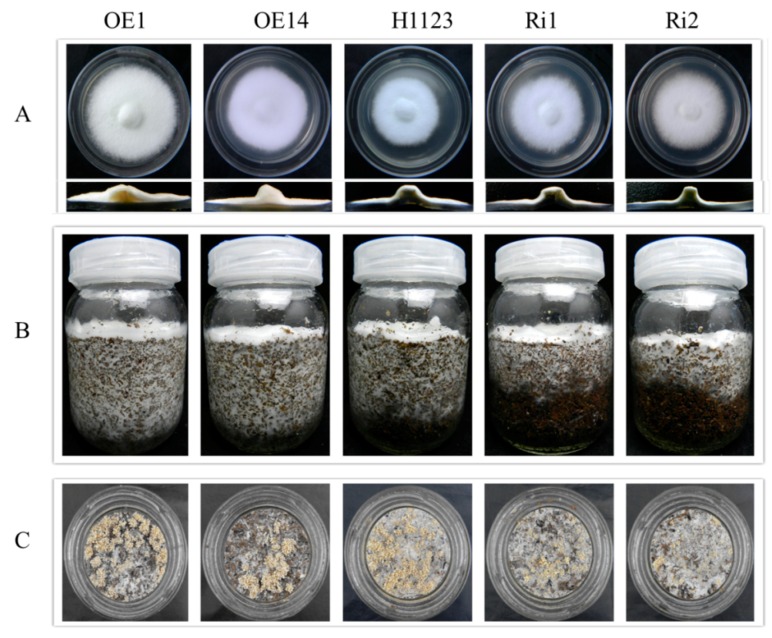
Phenotypic comparison of wild-type and *Fv-JRL1* mutants. (**A**) Colonies and cross-sections of wild-type and *Fv-JRL1* mutants after 5 days of incubation at 25 °C on potato dextrose agar (PDA) plates; (**B**) Growth after 22 days of incubation on composted substrate; (**C**) Fruiting trial.

To gain insight into the phenotypic alterations, transformants were incubated at 25 °C for 5 days. Experiments carried out on potato dextrose agar (PDA) plates showed that overexpression of *Fv-JRL1* resulted in an increase of growth and aerial hyphae compared to wild-type strain H1123. Further, cross-section of the two RNAi transformants indicated a reduction in the amount of hyphae in contrast to overexpression transformants (Figure 5A).

When grown on composted substrate, RNAi-silenced strains Ri1 and Ri2 produced fewer primordia than the wild-type (Figure 5C), exhibited retarded vegetative growth (Figure 5B), and delayed the formation of fruiting bodies. Wild-type *F. velutipes* H1123 needed 25 days of incubation to expand beyond the glass bottles, while Ri1 and Ri2 needed 29 days of incubation to reach to the bottom of bottles. The fruiting-body initiation appeared 15 days after mycelium stimulation by scratching the substrate surface off, while the fruiting body formation in Ri1 and Ri2 required 18 days when mycelium had covered the composted substrate surface after mycelium stimulation. In contrast, overexpressed strains OE1 and OE14 exhibited an increased growth rate (22 and 23 days, respectively, to reach to the bottom of bottles), producing fruiting bodies 13 days after removing the substrate surface.

## 3. Discussion

Lectins are also widely known as a class of carbohydrate-binding proteins that are important in a variety of biological processes, including cellular recognition [33], host-pathogen interactions, serum glycoprotein turnover, and innate immune responses [34]. The discovery of lectins was initially reported by Stillmark when studying proteinaceous hemagglutinating factor in *Ricinus communis* [35]. A variety of lectins have since been identified and purified in other organisms, including edible mushrooms [36], and they are widely used as reagents due to their medicinal properties. While lectins have been extensively studied in plants and animals, little research is available on their in vivo functions in mushrooms.

Previous studies indicate that mushroom lectins take part in the promoting differentiation of fruiting body primordia from the mycelia in *Agrocybe aegerita* [37]. In the present study, a lectin gene, *Fv-JRL1*, was identified based on the annotation of genome. Analysis of the deduced protein showed that Fv-JRL1 carried a conserved jacalin-like lectin domain and 33% identity to the jacalin-related lectin GFL (BAE43874.1) from *Grifola frondosa*, suggesting that Fv-JRL1 from the present study was in the same family of proteins. Phylogenetic analysis provided further evidence that the protein encoded by *Fv-JRL1* gene was a jacalin-related lectin.

*F. velutipes* has been appreciated not only as a useful model system for investigation of matrix degradation [38], but also as model fungal species for biochemical and physiological studies of formation and elongation of fruit body [23]. Fruiting body formation has recently attracted increased attention for many investigators. The present qRT-PCR analysis showed that *Fv-JRL1* expression was maximal at the primordia formation stage and then declined throughout the remaining stages of fruiting body development. These results suggested that *Fv-mJRL1* could be correlated with fruiting body formation in *F. velutipes*.

RNAi and overexpression, which can be applied for both down- and upregulation of target genes, have proven to be beneficial and useful tools for the manipulation of gene expression in fungi. With this in mind, RNAi and overexpression plasmids were constructed to contain the inverted-repeat sequences of the *Fv-JRL1* gene for silencing, and a fragment of the *Fv-JRL1* gene from ATG to TAG for overexpression, respectively. Integration of the *Fv-JRL1* silencing cassette led to a reduction in aerial mycelium and the fruiting body, consistent with the expression level of this gene, as well as delayed of fruiting body formation. Contrary to this, overexpression of *Fv-JRL1* resulted in an increase in aerial mycelium and promotion of fruiting body formation by reduction of the formation time. Taken together, these results clearly demonstrate that jacalin-related lectin gene *Fv-JRL1* in *F. velutipes* really does play an important regulatory role in the formation of aerial mycelium and fruiting body, thus providing insight into the physiological functions of lectins in vivo.

## 4. Materials and Methods

### 4.1. Strains, Vector, and Culture Conditions

Monokaryon strains W23 and dikaryon strain H1123 (a derivative of hybridization of monokaryon strains W23 and L11), provided by the Fujian Edible Fungi Germplasm Resource Collection Center of China [39]. For genomic DNA extraction using a modified CTAB (cetyltrimethyl ammonium bromide) approach [40], strain W23 was grown in liquid potato dextrose broth (PDB) at 25 °C for 10 days, and the mycelia were stored at −80 °C after being harvested.

*Escherichia coli* DH5α (Tiangen, Beijing, China) was employed for propagation of plasmids. The binary vector *pBHg-BCA1* was obtained from the Mycological Research Center of Fujian Agriculture and Forestry University.

### 4.2. Identification of Fv-JRL1 from F. velutipes

The sequence of the *Fv-JRL1* gene was obtained from the genome of *F. velutipes* strain W23, (GenBank Accession No. APHZ00000000; BioProject: 191864). For analysis of gene structure, all of the transcriptome reads at each developmental stage [39,41] were mapped to the full-length *Fv-JRL1* gene, as well as 2 kbp each of upstream and downstream sequences, using Zoom lite with a maximum of 40 mismatched base pairs [39,42]. In addition, comparison of the sequences of *Fv-JRL1* in W23 and L11 (GenBank Accession No. APIA00000000; BioProject: 191865) was done using DNAMAN.

### 4.3. Transcription Pattern Analysis of Fv-JRL1 Gene

*F. velutipes* strain H1123 was cultured on composted substrate prepared as described by Wang et al. [41]. Collection of mycelia and samples at different developmental stages was performed according to the method of Wang et al. [41], after which all samples were immediately frozen in liquid nitrogen.

Total RNA was isolated with an E.Z.N.A.^TM^ Plant RNA Kit (Omega, Stamford, CT, USA) in accordance with the manufacturer’s instructions. A NanoDrop ND-1000 Spectrophotometer (NanoDrop Technologies, Wilmington, DE, USA) was used to determine RNA concentration. Synthesis of cDNA was carried out using PrimeScript RT reagent Kit (Takara, Tokyo, Japan) according to the manufacturer’s instructions.

Quantitative real-time quantitative PCR (qRT-PCR) was conducted in triplicate on a CFX96 Real-Time PCR Detection System (Bio-Rad, Hercules, CA, USA). Bio-Rad iTaq TM Universal SYBR Green Supermix (Bio-Rad) was used in this study. Twenty-microliter reaction mixtures were prepared in accordance with manufacturer’s instructions using Bio-Rad iTaq^TM^ Universal SYBR Green Supermix (Bio-Rad). The qRT-PCR protocols were as follows: initial denaturation at 94 °C for 30 s; 40 cycles of 5 s at 95 °C, 30 s at 58 °C, and 65 °C for 5 s. Primer Premier 5.0 was applied for design of primers for *Fv-JRL1* and reference gene glyceraldehyde-3-phosphate dehydrogenase (*gapdh*) (Table S1) [39,43,44]. The transcript levels of target genes were calculated using 2^−ΔΔ*C*t^ method [45].

### 4.4. Construction of Gene Expression Plasmids

The binary vector *pBHg-BCA1* [44], encompassing the hygromycin B phosphotransferase (*hpt*) gene for hygromycin resistance and an *Agaricus bisporus* glyceraldehyde-3-phosphate dehydrogenase (*gpd*) gene promoter, was used for constructing an RNAi hairpin vector and overexpression vector.

**Figure 6:**
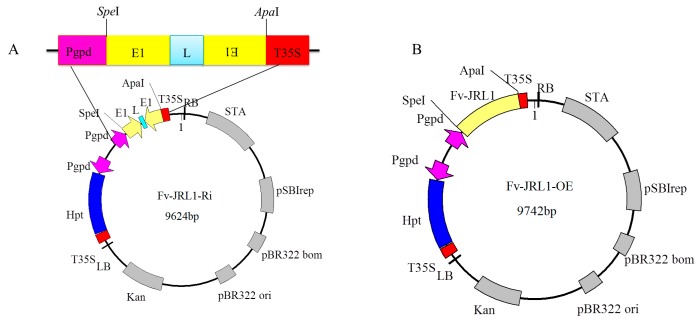
Binary vector *Fv-JRL1-RNAi* and *Fv-JRL1-OE*. *Pgpd*, *A. bisporus* glyceraldehyde-3-phosphate dehydrogenase gene promoter; *hpt*, hygromycin B phosphotransferase gene; *Kan*, kanamycin resistance gene; LB, left border; RB, right border. (**A**) Binary vector *Fv-JRL1-RNAi*; E1, exon1; L, linker; (**B**) binary vector *Fv-JRL1-OE*; Fv-JRL1, full-length of *Fv-JRL1* gene from *F. velutipes.*

A fragment of *Fv-JRL1* (GenBank Accession No. KU310976) from genomic DNA of *F. velutipes* strain W23 containing the region from base pairs 25 to 400, corresponding to the 5’ untranslated region, the first exon, and the first intron, was amplified by PCR with primers Fv-JRL1-S-F and Fv-JRL1-S-R. For reverse-orientation cloning, the 5’ untranslated region and the first exon of *Fv-JRL1* were amplified with primers Fv-JRL1-A-F and Fv-JRL1-A-R. PCR products were ligated into the *Spe*I and *Apa*I sites of plasmid *pBHg-BCA1* with the pEASY-Uni Seamless Cloning and Assembly Kit (Transgen Biotech, Beijing, China). Primer design and ligation were carried out according to the manufacturer’s instructions. A new plasmid, designated as *Fv-JRL1-RNAi*, was obtained (Figure 6) after sequence verification and digestion with *Spe*I and *Apa*I (Takara).

For overexpression assays, primers Fv-JRL1-F and Fv-JRL1-R, which introduced *Spe*I and *Apa*I recognition sites, respectively, were used to amplify the full-length *Fv-JRL1* gene (from ATG to TAG) from *F. velutipes* W23. The PCR product was digested with *Spe*I and *Apa*I, and ligated into the linearized plasmid *pBHg-BCA1* with the same two restriction endonucleases. The resulting plasmid *Fv-JRL1-RNAi*, containing the amplified full-length *Fv-JRL1* gene, was transferred into *E. coli* DH5α. The recombinant plasmid, designated as *Fv-JRL1-OE*, was subsequently identified using PCR and sequencing.

### 4.5. Eletrophoretic Mobility of Plasmids DNA: Liposomes Complexes at Different Ratios

To screen the optimal ratio between plasmid DNA and liposomes, a range of liposome volumes (Lipofectamine^TM^ 2000, Invitrogen, Carlsbad, CA, USA) was used for assessment of encapsulation of a constant amount of plasmid DNA. Briefly, 5 µL of *Fv-JRL1*-OE vector (200 ng/µL) was prepared prior to the addition of liposomes. A range of volumes of liposomes was then added, formulated as liposome/plasmid DNA (10 µL:1 µg, 9 µL:1 µg, 8 µL:1 µg, 7 µL:1 µg, 6 µL:1 µg, 5 µL:1 µg, 4 µL:1 µg, 3 µL:1 µg, 2 µL:1 µg, 1 µL:1 µg). The complexes were then incubated on ice for 30 min, followed by agarose gel electrophoresis to determine the optimal ratio of plasmid DNA to liposome by evaluating the ratio at which liposome-encapsulated DNA was distinguishable from plasmid DNA.

### 4.6. Fungal Transformation and Screening of Transgenic F. velutipes with PCR

The *Fv-JRL1*-OE and *Fv-JRL1*-RNAi plasmids were transferred to *F. velutipes* strain H1123 by liposome-mediated methods. Briefly, liposome/plasmid DNA complexes were prepared by combining liposomes and plasmid DNA at a ratio of 1 µL liposomes per 1 µg plasmid DNA after incubation on ice for 30 min. Mycelium homogenate of strain H1123 was obtained by grinding using a sterilized mortar, in which 1 mL of 0.6 M mannitol was added prior to grinding. The homogenate was then added rapidly to the liposome/plasmid DNA complexes and then incubated on ice for 30 min. Five times the total volume of liquid regeneration complete medium (RCM) (2.0 g tryptone, 2.0 g yeast, 0.5 g MgSO_4_·7H_2_O, 0.46 g K_2_HPO_4_, 20 g glucose, and 1 g KH_2_PO_4_ per liter) was added, and the resulting mixture was incubated at 25 °C and shaking at 150 rpm for 2 days. The mycelia were collected by centrifugation at 5000 rpm for 10 min, and incubated on PDA plates containing 25 μg/mL hygromycin B at 25 °C for 25 days. The hygromycin-resistant transformants, following five successive transfers to PDA plates containing 30 μg/mL hygromycin B, were assumed to be stable and selected for further investigation.

Genomic DNA from mycelia of the putative transformants was extracted as described previously [40]. Transformants were confirmed by PCR to detect the presence of the *hpt* gene using primers hpt-A and hpt-S, and the presence of sequences between the *gpd* gene promoter and the *Fv-JRL1* gene using primers GBT-F and Fv-JRL1-R for overexpression transformants, or the sequences between the *gpd* gene promoter and the first intron of *Fv-JRL1* using primers GBT-F and Fv-JRL1-S-R for RNAi transformants. PCR was conducted with rTaq Polymerase (Takara, Japan) using the following parameters: 94 °C for 5 min; 35 cycles of 94 °C for 45 s; 58 °C for 45 s; 72 °C for 1 min; and a final extension of 72 °C for 10 min.

### 4.7. RNA Extraction and qRT-PCR

*F. velutipes* transformants and wild-type strain H1123 were grown in PDB medium at 25 °C and shaking at 150 rpm for 15 days. The mycelia were harvested, and then frozen in liquid nitrogen. Isolation of total RNA and qRT-PCR were conducted as described above.

### 4.8. Phenotype of Mutants

For morphology of the colonies, 5 mm mycelial pieces were placed onto the PDA plates and grown in the dark at 25 °C for 5 days. The morphology of the colonies was observed daily, and then photographed on day 5 after incubation. In addition, fruiting trials for transformants and wild-type strains were carried out in glass bottles containing 180 g composted substrate prepared as described by Wang et al. [41]. Experiments were replicated three times.

## Supplementary Materials

Supplementary materials can be found at www.mdpi.com/1422-0067/17/11/1884/s1.
