# Supplementary material for: A Jacalin-Related Lectin Regulated the Formation of Aerial Mycelium and Fruiting Body in Flammulina velutipes
Source: Int J Mol Sci. 2016 Nov 28;17(12):1884. doi: 10.3390/ijms17121884 (PMC5187758; doi:10.3390/ijms17121884)
Supplement: Supplementary file 1 [file ijms-17-01884-s001.pdf]

Supplementary Materials: A Jacalin-Related Lectin Regulated the Formation of Aerial Mycelium and Fruiting Body in *Flammulina velutipes*

Yuan-Ping Lu, Ren-Liang Chen, Ying Long, Xiao Li, Yu-Ji Jiang and Bao-Gui Xie

Table S1. Primers used in this study.

Primer	Nucleotide Sequence (5′–3′)
gapdh-F	CCTCTGCTCACTTGAAGGGT
gapdh-R	GCGTTGGAGATGACTTTGAA
Fv-JRL1-F	GCCAGCCTTCTCGGTAGC
Fv-JRL1-R	CGTAGACACGGTCGCCAG
Fv-JRL1-F-*Spe*I	GGACTAGTCCTAAACTCTCCATTACTCTCAGC
Fv-JRL1-R-*Apa*I	GAGGGCCCCATGATTTAGGCAAGCAAAATAG
Fv-JRL1-S-F	TCTACACACAACAACTTATCGCTATCCCAAAACAACATCGG
Fv-JRL1-S-R	GGATTACAGACCCTTCAACGACTGGTGTTGAGTATGGCGTTAGG
Fv-JRL1-A-F	CATGCCAATTCTAGAGGGCCGCTATCCCAAAACAACATCGG
Fv-JRL1-A-R	CCTAACGCCATACTCAACACCAGTCGTTGAAGGGTCTGTAATCC
Hpt-F	CTATTCCTTTGCCCTCGG
Hpt-R	ATGAAAAAGCCTGAACTCACC
GBT-F	CCCAGGCTTTACACTTTAT

Restriction sites are underlined.

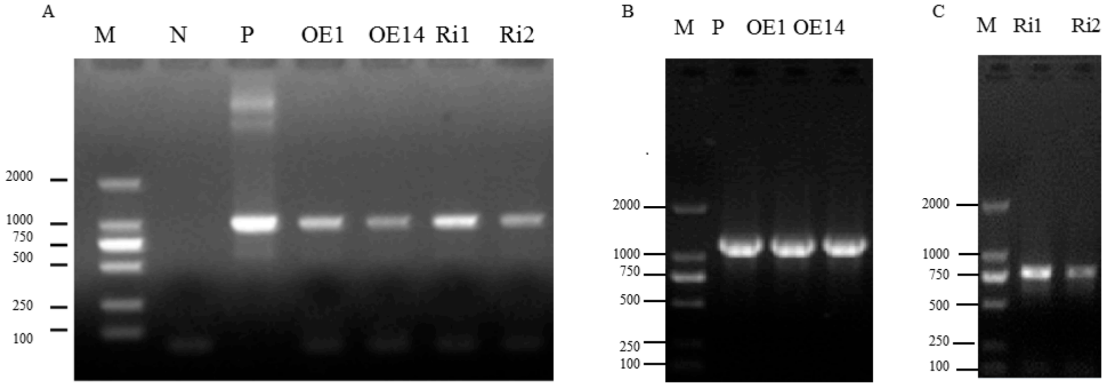

Figure S1. The identification of Fv-JRL1 putative transformants using PCR, M: Maker DL2000; P: plasmid *pBHg-Fv-JRL*-OE; N: wild-type strain H1123. (**A**) Presence of the hygromycin B resistance gene (*hpt*) in transformants. Primers Hpt-F and Hpt-R were adapted; (**B**) Presence of Pgpd and the full-length *Fv-JRL1* gene. PCR with specific primers of *Pgpd* and the full-length *Fv-JRL1* gene was carried out with GBT-F and *Fv-JRL1*-R; (**C**) Presence of *Pgpd* and a 376 bp fragment cloned in forward orientations in the silencing cassette. PCR with specific primers GBT-F and *Fv-JRL1*-S-R was performed with RNAi transformant genomic DNA.
